# A novel functional water significantly modulates the gut microbiota and decreases the basal level of inflammation in mice

**DOI:** 10.3389/fnut.2025.1718745

**Published:** 2026-01-12

**Authors:** Yinghui Men, Lixia Yue, Mingchao Zhang, Bing Wang, Weihai Ying

**Affiliations:** 1 School of Biomedical Engineering and Med-X Research Institute, Shanghai Jiao Tong University, Shanghai, China; 2 School of Medical Instrument and Food Engineering, University of Shanghai for Science and Technology, Shanghai, China; 3 State Key Laboratory of Systems Medicine for Cancer, Shanghai Cancer Institute, Renji Hospital, School of Medicine, Shanghai Jiao Tong University, Shanghai, China

**Keywords:** functional water, beneficial gut bacterium, harmful gut bacterium, low-grade chronic inflammation, decrease inflammation

## Abstract

**Background:**

Accumulating evidence has identified the gut microbiota as a critical regulator of biological processes such as immune homeostasis, and its dysbiosis has been implicated in the pathogenesis of numerous diseases. Current strategies for modulating the gut microbiota remain limited by several challenges: low colonization efficiency (probiotics), interindividual variability in host response (prebiotics), and safety concerns (antibiotics).

**Methods:**

We investigated the effects of a physically processed, additive-free purified water (Koishio water, KW) on gut microbiota composition and basal inflammatory status in healthy mice.

**Results:**

First, KW drinking significantly increased the diversity and richness of the gut microbiota of the mice, including increased abundance of Verrocomicrobiota and decreased abundance of Proteobacteria; Second, KW drinking significantly increased the abundance of several beneficial genera of the gut bacterium (*Akkermansia, Faecalibaculum, Ligilactobacillus*, and *Muribaculum*) and significantly decreased the abundance of several harmful genera of the gut bacterium (*Clostridioides, Citrobacter, Escherichia-Shigella*, and *Clostridium Innocuum group*); and third, functional prediction suggested enrichment of microbial pathways related to mucosal barrier integrity, host metabolic regulation, and antioxidative capacity. Moreover, KW significantly decreased the basal level of three major pro-inflammatory factors, including IL-1β, IL-6, and TNF-α, which are widely recognized as biomarkers of subclinical low-grade chronic inflammation.

**Conclusion:**

These findings demonstrate that KW, characterized by affordability, safety, and suitability for daily consumption, may serve as a novel non-pharmacological intervention to beneficially modulate gut microbiota composition and reduce basal inflammatory levels under non-pathological conditions.

## Introduction

A large number of studies have indicated that alterations of gut microbiota are novel mechanisms underlying a number of diseases, contributing to nutrient digestion, immune homeostasis, and defense against pathogenic colonization (1–7). Numerous studies have also indicated that alterations of gut microbiota are causative to multiple diseases, including metabolic diseases such as Type II diabetes, diseases of the digestive system such as inflammatory bowel disease (IBD), neuropsychiatric diseases such as autism, and age-associated diseases (2–5). These studies highlight the crucial role of maintaining microbial homeostasis in promoting host health and preventing disease.

The composition and function of the gut microbiota are influenced by a wide range of intrinsic and environmental factors, such as genetics, age, diet and medications (8, 9). Among these, diet has been extensively studied for its potent and rapid effects on the gut microbiome, with components such as dietary fiber, saturated fats, and polyphenols known to influence microbial community structure, short-chain fatty acid production, and immune regulation (10, 11). Despite the increasing interest in microbiota-targeted therapies, current strategies for modulating the gut microbiota remain limited by several challenges. Probiotics often exhibit low colonization efficiency and highly variable responses across individuals (12). Prebiotics, while promising, are associated with high production costs and a lack of robust clinical evidence (13). Antibiotic-based approaches, though effective in altering microbial communities, raise serious concerns regarding safety, resistance, and ethical considerations (13). Therefore, it is of significant scientific and medical significance to find novel approaches that can enhance the healthy state of gut microbiota.

The development of drinking water has played a pivotal role in improving human health and longevity. According to Cutler and Miller (14), water filtration and chlorination in U.S. cities between 1900 and 1940 nearly doubled life expectancy, from just over 30 to nearly 70 years. Water is implicated in all aspects of life activities and can also affect the composition of gut microbiota in the host. It has been reported that drinking water source is associated with distinct signatures of the gut microbiota in US and UK populations (15). It has also been reported that the pH change in drinking water affected the incidence of diabetes and the composition of gut microbiota in diabetes-prone non-obese diabetic mice (16). In addition, many kinds of natural mineral water have been reported to reduce intestinal inflammation and regulate metabolism to reduce obesity risk, via modulating gut microbiota (17, 18). Due to the critical significance of gut microbiota for the healthy state of people, it is of great interest to further determine the effects of various types of drinking water on the gut microbiota.

Our preliminary studies have suggested that Koishio technology-produced water (KW) has significant antioxidant capacity in H_2_O_2_-treated cell culture studies (19). Since oxidative stress may produce significant effects on gut microbiota that can affect the inflammatory processes, we hypothesized that KW drinking might significantly affect gut microbiota, thus affecting the basal level of inflammation. In this study, we used a mouse model to test this hypothesis (20). Our study has found that KW drinking produced significant beneficial effects on the gut microbiota and decreased the basal level of inflammation in mice.

## Experimental section

### Materials

All chemicals were purchased from Sigma (St. Louis, MO, USA) except where noted. KW (Ultra Small Nanobubbles Water) was purchased from Shanghai Koishio Food Industry Co. (Shanghai, China). Commercial purified water was purchased from China Resources Beverage (Shenzhen, China), and commercial mineral water was purchased from Nongfu Spring (Hangzhou, China) and Shenzhen Ganten Food & Beverage Co., Ltd (Shenzhen, China).

### KW manufacturing process

The raw water was first filtrated by activated carbon and quartz sand to remove suspended solids and sticky particles, followed by a 5 μm pore-size filtration membrane. After that, the primary reverse osmosis separated the solvent by pressure difference. The water was sterilized by ultraviolet irradiation, and then further impurities were removed through secondary reverse osmosis. Finally, nano-bubbles were produced by a composite physical approach, and the pure water was secondary sterilized by high temperature.

### Detection of heavy metal elements

The presence of heavy metal elements in water was detected by water quality test strips (Qingshui 16 in 1 Water Quality Test Strips, Foshan, China). The strips were immersed in the respective water samples, followed by being placed on a white background for color evaluation.

### Detection of total dissolved solids in multiple water

The total dissolved solids (TDS) detector was purchased from Deli Group Co. (Ningbo, China). Each type of water was divided into six replicates and detected by a TDS detector.

### Detection of nano-bubbles in water

The number density and size of nano-bubbles were determined via nanoparticle tracking analysis (NTA) system (NS300, Malvern, UK). The results were analyzed by NTA 3.4 software (Malvern). In addition, the size of nano-bubbles was also determined by dynamic light scattering (DLS, nano-ZS90, Malvern) (21).

### Animal model of KW drinking

All animal experiments were conducted according to the animal protocols approved by the Bioethics Committee, Shanghai Jiao Tong University School of Biomedical Engineering. All animals were maintained under a 12/12 h light/dark cycle, with a constant temperature of 23 ± 2 °C and 40–70% humidity. Male C57BL/6 mice were purchased from SLARC Laboratory (Shanghai, China) at 18–24 g weight, housed in a specific pathogen-free facility, and orally administered with ddH_2_O or KW for 17 days. The fresh drinking water was replaced every 3 days. Mice were inspected daily, and both body weight and water consumption were recorded. On the 17th day, animals were anesthetized by injection of 200 mg/kg tribromoethanol, and fecal samples were collected for determinations of gut microbiota. Finally, animals were euthanized by anesthetized and cervical dislocation. Colon tissues were collected to determine cytokine levels. Other organs were weighed on the 17th day.

### Gut microbiome 16S rRNA gene sequencing

To elucidate the structural and functional properties of the intestinal microbiota, high-throughput sequencing was employed to analyze the V3-V4 variable region of the 16S rRNA gene in the feces. First, genomic DNA from the gut microbiota was extracted using the E.Z.N.A^®^ soil DNA Kit (Omega Bio-tek, Norcross, GA, USA) (22). The concentrations and purity of the extracted DNA were quantified using a NanoDrop spectrophotometer (ThermoFisher, Waltham, MA, USA). The DNA quality was assessed by electrophoresis on a 1% agarose gel. Second, the V3–V4 regions of the 16S rRNA gene were amplified with the primer pair 338F (5′-ACTCCTACG GGAGGCAGCAG-3′) and 806R (5′-GGACTACHVGGGTWTCTAAT-3′) by a thermocycler PCR system (GeneAmp 9700, ABI, Foster, CA, United States). After PCR amplification, the product was purified using the AxyPrep DNA Gel Extraction Kit (AP-GX-250G, Axygen Biosciences, USA), and the quality of the amplicon was measured with a QuantusTM Fluorometer (Promega, Madison, USA). Amplicon sequencing was conducted on the Illumina MiSeq PE300 system (Promega, San Diego, USA).

### Calculations of diversity and richness of the gut microbiota of mice

For data analysis, raw sequencing data were quality-filtered using fastp (version 0.21.0) and merged by FLASH (version 1.2.7), followed by adaptor sequence reading and trimming of low-quality bases (quality score less than Q20). In addition, short, truncated reads (less than 50 bp) containing ambiguous nucleotides were discarded. The paired-end reads were merged based on the maximum mismatch ratio of 0.2 with a minimum overlap of 10 bp in the overlapping region. The merged data were used for clustering into operational taxonomic units (OTUs) at a 97% similarity threshold. The sequences were then aligned against the SILVA138 reference database with a minimum confidence score of 0.7 for taxonomic assignment. Richness was calculated according to the OTUs and reference database (23). Alpha diversity was estimated using the Chao1 and Shannon indices. Principal coordinates analysis (PCoA) based on Bray-Curtis matrices with statistical significance determined by permutational multivariate analysis of variance (PERMANOVA) was conducted to assess the differences in beta diversity between groups (23). Finally, the Linear Discriminant Analysis Effect Size (LefSe) analysis was used to determine the noteworthy microbial taxa.

### Determinations of the mRNA levels of IL-1β, IL-6, and TNF-α

The real-time PCR assays were conducted as described previously (24, 25). Total RNA was extracted by FastPure Cell/Tissue Total RNA Isolation Kit (Vazyme, Nanjing, China) from collected BV2 cells and colon tissues. Five hundred ng of total RNA was reverse transcribed to cDNA using HiScript IV 1st Strand cDNA Synthesis Kit (Vazyme, Nanjing, China). The parameters set for reverse transcription were as follows: 50 °C for 5 min, then 85 °C for 5 s. Quantitative RT-PCR was performed by using ChamQ Universal SYBR qPCR Master Mix (Vazyme, Nanjing, China) and the following primers:

IL-1β primers: sense 5′-TTGACGGACCCCAAAAGATG-3′, anti-sense 5′-AGAAGGTGCTCATGTCCTCA-3′;

IL-6 primers: sense 5′-TCTATACCACTTCACAAGTCGGA-3′, anti-sense 5′-GAATTGCCATTGCACAACTCTTT-3′;

TNF-α primers: sense 5′-TCTCATCAGTTCTATGGCCC-3′, anti-sense 5′-GGGAGTAGACAAGGTACAAC-3′;

GAPDH primers: sense 5′-CAACTTTGGCATTGTGGAAGG-3′, anti-sense 5′-ACACTTTGGGGGTAGGAACAC-3′.

PCRs were performed according to the following procedure: after denaturing at 95 °C for 10 min, 40 cycles of reactions were conducted according to the following procedures of reactions: 95 °C for 10 s, 60 °C for 10 s, and 72 °C for 10 s. The data were analyzed by using the comparative threshold cycle method, and results were expressed as fold difference normalized to the level of GAPDH mRNA.

### Immunohistochemical analysis

The colon tissue section was incubated with IL-1β (GB11113-50, Wuhan, Servicebio), IL-6 (GB11117-50, Wuhan, Servicebio), and TNF-α (GB11188-50, Wuhan, Servicebio) antibodies overnight at 4 °C, respectively (26). Subsequently, the slides were incubated with secondary antibodies for 1 h at room temperature. Finally, the slides were washed in phosphate buffer solution (PBS) 3 times, followed by the meticulous introduction of 3,3′-Diaminobenzidine (DAB) chromogenic solution to visualize the targeted cells.

### Hematoxylin and Eosin (H&E) staining

The collected colon tissues were fixed in 4% paraformaldehyde solution overnight, followed by dehydration, embedded in paraffin, and cut into sections of 5 μm (27). The paraffin sections were stained with a hematoxylin and eosin (H&E) staining kit (Beyotime, Shanghai, China).

### Determination of mice basal healthy level

The weight of mice was measured and recorded every day. The relative weight was calculated by the weight of the day over the initial weight. The organ indexes were calculated by the organ weight over the body weight after mice were sacrificed (27).

### Cell culture models

The murine microglia cell line BV2 cells were purchased from the Chinese Academy of Sciences Cell Bank. BV2 cells were grown in KW-based Dulbecco's modified Eagle medium (DMEM) or ddH_2_O-based DMEM, both of which were supplemented with 10% heat-inactivated fetal bovine serum (FBS, A5669701, Gibco), 100 U/mL penicillin, and 0.1 mg/mL streptomycin (15140122, Gibco) at 37 °C in a humidified 5% CO_2_ atmosphere.

### Production of cell culture media

The culture media were prepared according to the manufacturer's instructions. In brief, 13.5 g of DMEM powder (12800017, Gibco) was added to 950 mL of sterilized ddH_2_O or KW. Subsequently, 1 N HCl was used to adjust the pH to around 4.1–4.2, followed by transferring 47.5 mL of media to a 50 mL centrifuge tube. Approximately 1.5 mL of 7.5 % NaHCO_3_ solution was added to each 50 mL tube to adjust the pH to 7.2–7.4. Finally, sterile syringe filters with a 0.22 μm pore size (BE-PES-22, Biosharp) were used to filter the culture media.

### Determinations of cell viability

Cell viability was quantified by measuring the intracellular lactate dehydrogenase (LDH) activity of the cells (28). Intracellular LDH activity was determined by measuring the LDH activity of cell lysates. Cell lysates were prepared through the following procedures: Cells were lysed for 15 min in lysing buffer containing 0.04% Triton X-100, 2 mM HEPES, and 0.01% bovine serum albumin (pH 7.5). For LDH activity assays, 50 μL of cell culture medium or 50 μL of cell lysates was mixed with 150 μL of 500 mM potassium phosphate buffer (pH 7.5) containing 0.34 mM NADH and 2.5 mM sodium pyruvate. The A340nm changes were monitored over 90 s The percentage of cell survival was calculated by normalizing the LDH values of samples to the LDH activity measured in the lysates of control (wash only) culture wells.

Cell Counting Kit-8 (CCK-8, Beyotime, China) was also used to determine the cell viability (29). In short, cells were cultured in different media. After removing the media, fresh DMEM with CCK-8 was mixed, followed by adding it to the cells. The CCK-8 media was determined at 450 nm absorbance, after 1 h of incubation.

### Determinations of intracellular ROS levels

For the Dichlorofluorescin (DCF) assay, 2,7-dichlorofluorescin diacetate (DCFH-DA, Beyotime, China), a reactive oxygen species-specific fluorescent probe, was used to measure total intracellular ROS levels (30). After BV2 cells were cultured in either regular cell culture media or KW cell culture media for 24 h, the cells were exposed to 1 mM H_2_O_2_ prepared in Minimum Essential Medium (MEM, 11095080, Gibco) for 1 h. After the treatment, the cells were cultured in regular cell culture media or KW cell culture media for 18–24 h. The cells were incubated with 20 μM DCFH-DA dissolved in DMEM without FBS for 30 min at 37 °C in an incubator. After 3 times washes with PBS, the cells were analyzed by flow cytometry (FACS Aria; Becton Dickinson, Heidelberg, Germany) to detect the mean fluorescence intensity (MFI) with an excitation wavelength of 488 nm and emission wavelength of 525 nm.

### Nitric oxide assay

A NO Test Kit (Beyotime, Jiangsu, China) was used to determine the level of NO, according to the manufacturer's instructions Cell culture medium, Griess reagent I, and Griess reagent II were mixed, followed by reading the absorbance at 540 nm using a microplate reader (30). The result was calculated by normalizing the concentrations of nitrite in the samples to concentrations of the standards and concentrations of proteins, which were determined by the bicinchoninic acid (BCA) assay.

### Statistical analysis

The R (version 4.1.3) and GraphPad 10 were used to perform general statistical analysis and visualize results via packages vegan (v2.6-4), phyloseq (v1.38.0), tidyverse (v1.3.2), ggpubr (v0.5.0), ComplexHeatmap (v2.10.0), and corrplot (v0.92). Data were presented as mean ± SEM and analyzed by two-tailed Student's *t*-test. *P*-values less than 0.05 were considered statistically significant.

## Results

### KW was pure water only containing ultra-small nanobubbles

**Figure 1:**
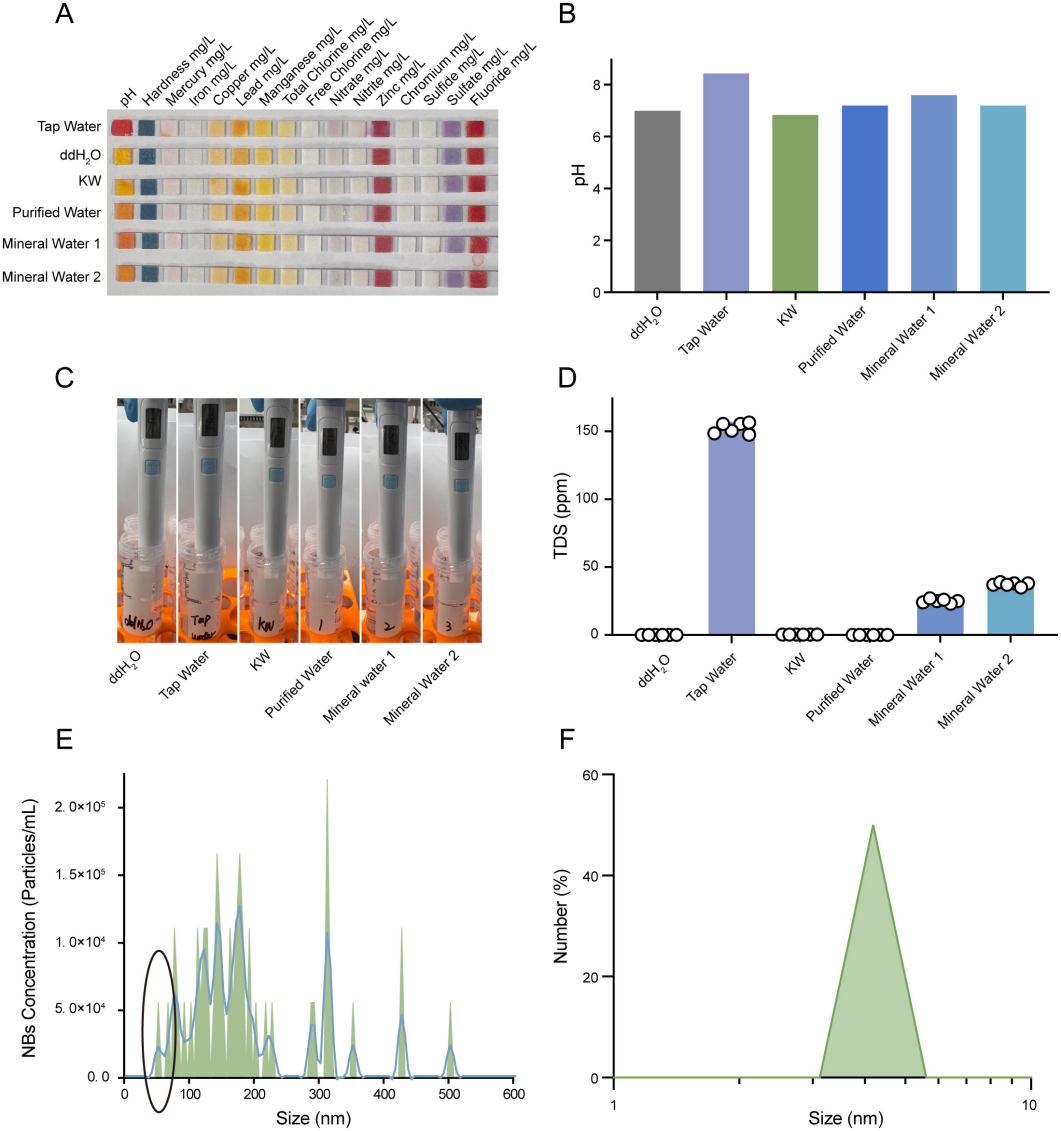
KW was ultrapure water with ultra-small nanobubbles. **(A, B)** Representative images and quantitative analysis of pH and heavy metal element detection, including ddH_2_O, tap water, KW, commercial purified water, and commercial mineral water. **(C, D)** Representative images and quantitative analysis of TDS detection of ddH_2_O, tap water, KW, commercial purified water, and commercial mineral water. **(E, F)** The number density and size of nano-bubbles were determined via NTA system and DLS instrument.

We studied the preparation process of KW and tested its various physical properties. KW was produced by multistage filtration, sterilization, and composite physical approaches (Supplementary Figure S1A). The heavy metal elements detection confirmed the absence of detectable heavy metal elements in KW, and the pH of KW was approximately neutral (Figures 1A, B and Supplementary Table S1). In addition, the TDS detection indicated that the KW contained no detectable impurities, compared with some commercial water (Figures 1C, D and Supplementary Table S2). The NTA system and DLS instrument both showed strong evidence that there were plenty of ultra-small nanobubbles in KW with a size less than 50 nm (Figures 1E, F). Collectively, these results indicate that KW is an ultrapure form of water characterized by the exclusive presence of ultra-small nanobubbles.

### The safety of KW treatment both *in vitro* and *in vivo*

**Figure 2:**
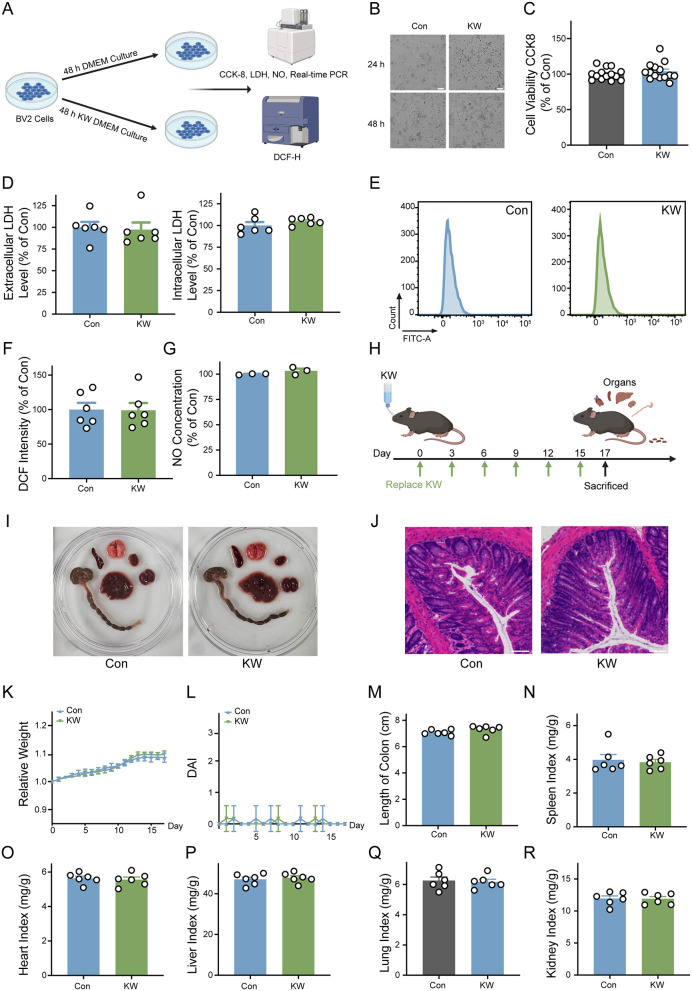
The safety of KW treatment both *in vitro* and *in vivo*. **(A)** Schematic diagram of the KW treatment cell model. **(B)** Representative diagrams of the cell under the bright field, scale bar = 100 μm. **(C, D)** The cell viability was determined by CCK8, extracellular LDH level, and intracellular LDH level. *N* = 14 for CCK8, N = 6 for the LDH test. **(E, F)** The representative diagrams and quantitative analysis of the FACS-based assay using DCFH as a ROS probe. *N* = 6. **(G)** The cellular NO level was determined by reading the absorbance at 540 nm using a microplate reader. *N* = 3. **(H)** Schematic diagram of the KW drinking animal model. **(I)** The representative images of various organs. **(J)** H&E staining of colon tissue. Scale bar = 50 μm. **(K, L)** The relative body weight and disease activity index (DAI) of mice. *N* = 6. **(M–R)** The length of the colon, spleen index, heart index, liver index, lung index, and kidney index of mice. Organ index was calculated by organ weight over body weight. *N* = 6. **(A, H)** were created with BioRender.com.

The safety of KW was determined both *in vitro* and *in vivo*, by determining the cell viability, oxidative stress of the cell, and different organ index of mice. The BV2 cells were cultured with normal DMEM or KW DMEM for 48 h (Figure 2A). The cell brightfield images and CCK8 test showed there was no difference in cell viability between the Con group and KW group (Figures 2B, C). Extracellular and intracellular LDH activities also indicated that the two groups showed equal cell viability (Figure 2D). The DCFH staining and NO test showed that KW did not affect the basal level of oxidative stress (Figures 2E–G). The animal water-drinking model was used to study the influence of KW on safety and gut microbiota. Seventeen days after mice started drinking ddH2O or KW, the organ indices of the mice were analyzed (Figure 2H). The ddH_2_O was used to feed the control group to establish a standardized and chemically defined water-drinking control that allows direct comparison with KW under identical and controlled experimental conditions. The representative images of various organs and H&E staining of the colon showed the safety of KW for mouse (Figures 2I, J). Further analysis also indicated that KW drinking did not affect the body weight, disease activity index (DAI), length of colon, spleen index, heart index, liver index, lung index, and kidney index (Figures 2K–R). The criteria for DAI scores were given in Supplementary Table S3. These results collectively demonstrate that KW consumption is safe and well-tolerated under the conditions tested.

### KW drinking significantly increased the diversity and richness of the gut microbiota of the mice

**Figure 3:**
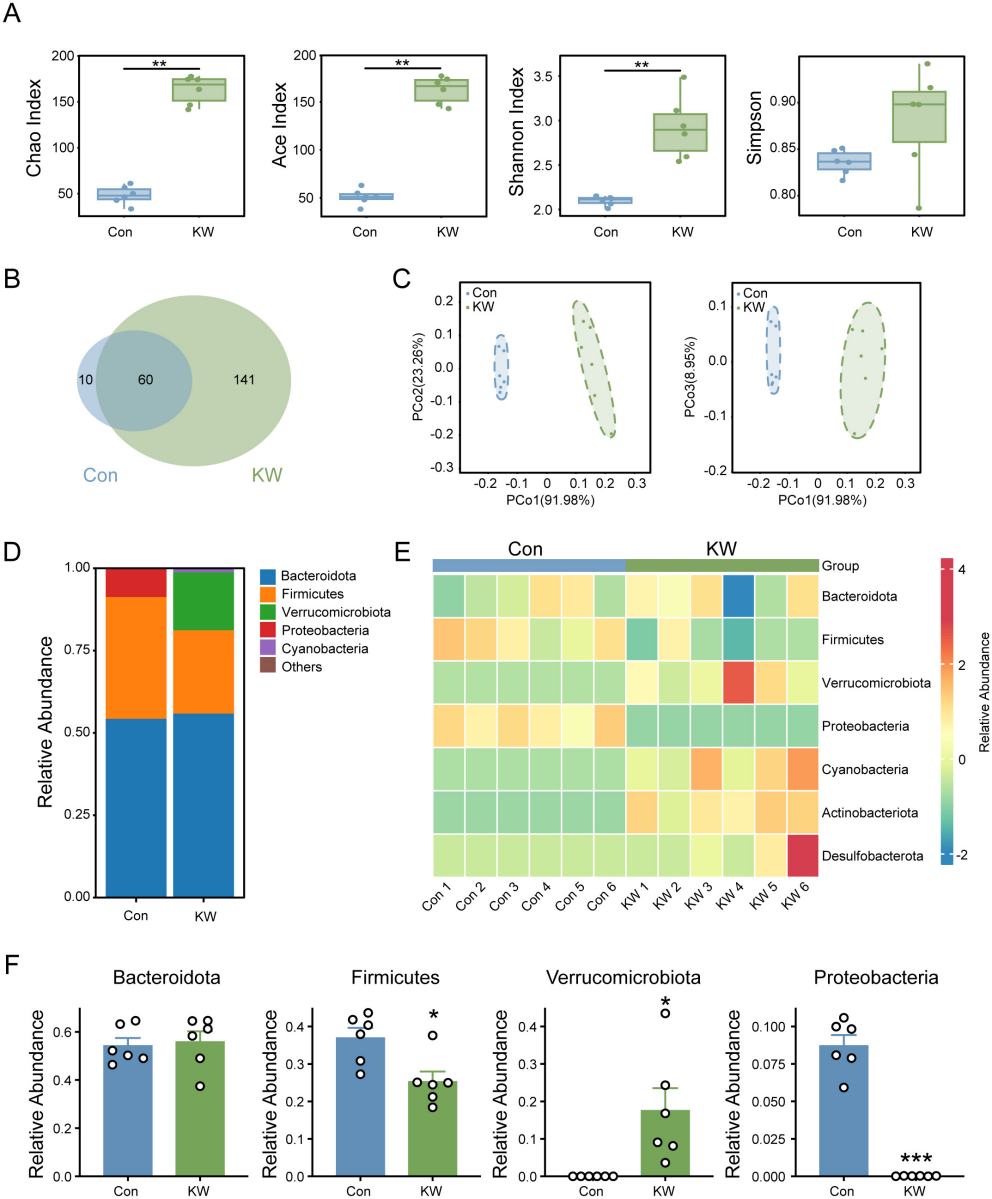
KW drinking significantly changed the abundance of several phyla in the gut microbiota of the mice. **(A)** Alpha diversity analysis on Chao1 index, Ace index, Shannon index, and Simpson index showed a marked increase in species richness and evenness. *N* = 6. **(B)** The Venn diagram illustrates that the KW group exhibited a higher number of unique OTUs compared to the Con group. *N* = 6. **(C)** Beta diversity analysis using Principal Coordinates Analysis (PCoA) showed a distinct clustering of microbial communities between the two groups. *N* = 6. **(D, E)** The distribution of several phyla in the gut microbiota of the mice. *N* = 6. **(F)** The quantitative analysis of several phyla in the gut microbiota of the mice. *N* = 6. *p < 0.05, **p < 0.01, ***p < 0.001.

The alpha diversity analysis, including Chao1 index, Ace index, Shannon index, and Simpson index, indicated a marked increase in microbial diversity and evenness in the KW group (Figure 3A). The Venn diagram illustrates that the KW group exhibited a higher number of unique OTUs compared to the Con group (Figure 3B), indicating a notable increase in species richness in the KW group. Moreover, beta diversity analysis using PCoA showed a distinct clustering of microbial communities between the two groups (Figure 3C), suggesting that the KW drinking induced a substantial shift in the composition of the gut microbiota.

### KW drinking significantly changed the relative abundance of several phyla in the gut microbiota of mice

At the phylum level, KW drinking significantly shifted the microbial composition (Figures 3D, E). The KW drinking significantly increased the abundance of Verrucomicrobiota, while the KW drinking also led to a significant decrease in the abundance of Firmicutes and Proteobacteria (Figure 3F). These results indicate that KW induced marked compositional changes in the gut microbiota at higher taxonomic levels.

### KW drinking significantly increased the abundance of several beneficial genera of the gut bacterium and decreased the abundance of several harmful genera of the gut bacterium in mice

**Figure 4:**
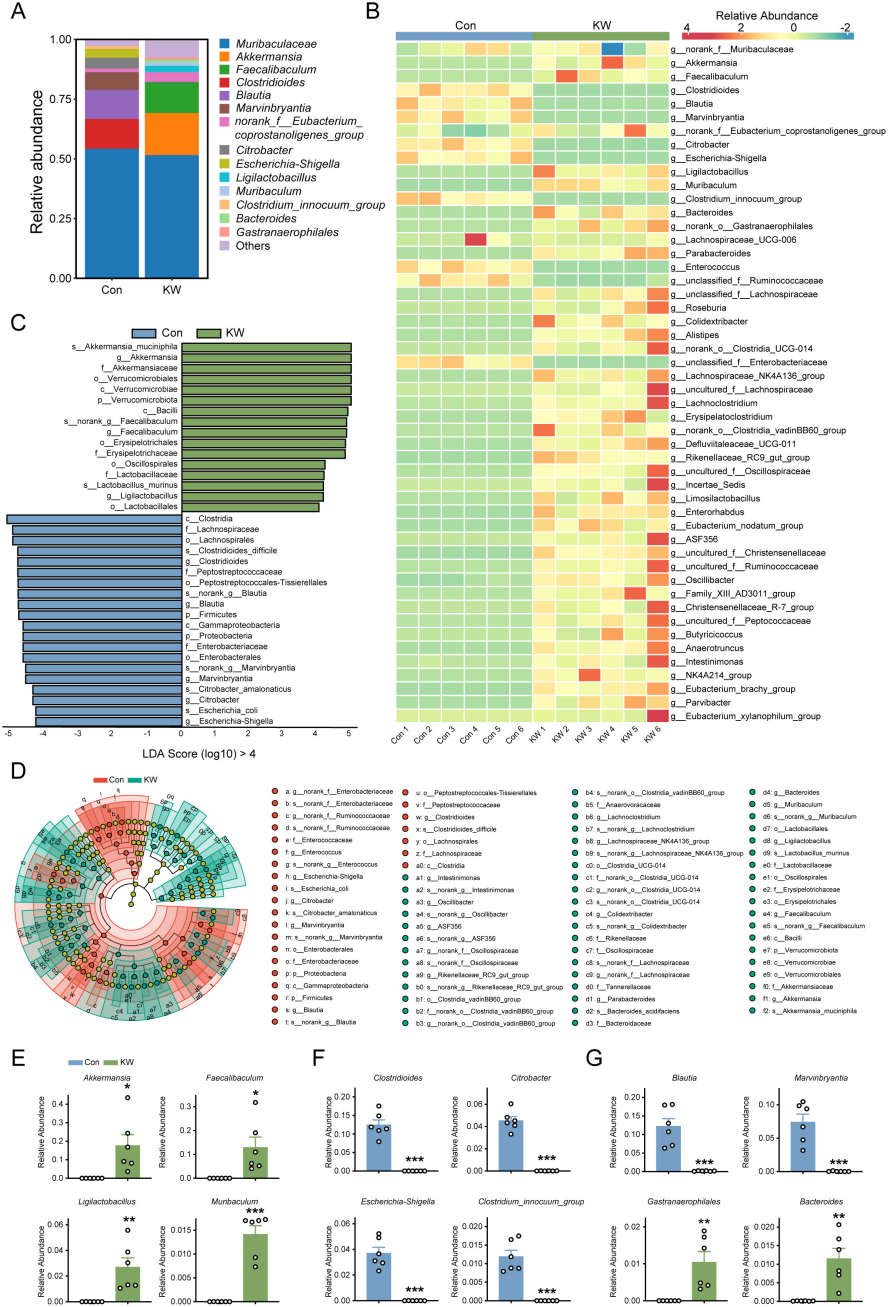
KW drinking significantly changed the abundance of several genera in the gut microbiota of the mice. **(A, B)** The distribution of several genera in the gut microbiota of the mice. *N* = 6. **(C)** The Linear Discriminant Analysis (LDA) score of the gut bacteria with an LDA Score (log10) greater than 4, indicating a higher abundance in the corresponding group. *N* = 6. **(D)** Identification of typical taxa among the two groups by Linear Discriminant Analysis Effect Size (LEfSe) analysis. *N* = 6. **(E)** The quantitative analysis of several beneficial genera in the gut microbiota of the mice. *N* = 6. **(F)** The quantitative analysis of several harmful genera in the gut microbiota of the mice. *N* = 6. **(G)** The quantitative analysis of several other genera with significant differences in the gut microbiota of the mice. *N* = 6. *p < 0.05, **p < 0.01, ***p < 0.001.

Genus-level analysis revealed that KW intake produced pronounced shifts in the abundance of several bacterial taxa (Figures 4A, B). Linear Discriminant Analysis (LDA) was used to determine the most remarkable differences in the intestinal bacteria taxa. The LDA score showed that the following gut bacteria with LDA Score (log_10_) greater than 4 were significantly enriched in the KW group (Figure 4C): s_Akkermansia_muciniphila, *g_Akkermansia*, f_Akkermansiaceae, o_Verrucomicrobiales, c_Verrucomicrobiae, p_Verrucomicrobiota, c_Bacilli, *s__norank_g__Faecalibaculum, g__Faecalibaculum*, o__Erysipelotrichales, f__Erysipelotrichaceae, o__Oscillospirales, f__Lactobacillaceae, s__Lactobacillus_murinus, *g__Ligilactobacillus*, o__Lactobacillales. Otherwise, there were some gut bacteria were significantly enriched in the Con group (Figure 4C): c_Clostridia, f_Lachnospiraceae, o_Lachnospirales, s_Clostridioides_difficile, *g_Clostridioides*, f_Peptostreptococcaceae, o_Peptostreptococcaceae_Tissierellales, *s_norank_g_Blautia, g_Blautia*, p_Firmicutes, c_Gammaproteobacteria, p_Proteobacteria, f_Enterobacteriaceae, o_Enterobacterales, *s_norank_g_Marvinbryantia, g_Marvinbryantia*, s_Citrobacter_amalonaticus, *g_Citrobacter*, s_Escherichia_coli, *g_Escherichia-Shigella*. Next, to identify the specific bacterial taxa after KW drinking, linear discriminant analysis effect size (LEfSe) analysis was employed. The cladogram indicated that the representative structure of the gut microbiota and their predominant bacteria in the Con group and KW group (Figure 4D). There were 27 microbial taxa dominant in the Con group and 52 microbial taxa dominant in the KW group (Figure 4D).

The important abundance change of various genera of the gut bacterium of the mice was summarized in Figures 4E, F. Compared with the abundance of various genera of the gut bacterium of the mice that drank regular water, there was increased abundance of several beneficial genera of the gut bacterium of the mice that drank KW, such as *Akkermansia, Faecalibaculum, Ligilactobacillus* and *Muribaculum* (Figures 4E), while there was decreased abundance of several harmful genera of the gut bacterium such as *Clostridioides, Citrobacter, Escherichia-Shigella* and *Clostridum_innocuum_group* (Figure 4F). The KW drinking was also found to significantly decrease the abundance of *Blautia* and *Marvinbryantia* of the gut bacterium (Figure 4G). Moreover, the KW drinking was found to significantly increase the abundance of *Gastranaerophilales* and *Bacteroides* of the gut bacterium (Figure 4G). These bacteria belong to the genus of gut bacteria that can produce either beneficial or harmful effects under different conditions.

### KW drinking significantly changed the metabolic pathways of the gut microbiota in mice

**Figure 5:**
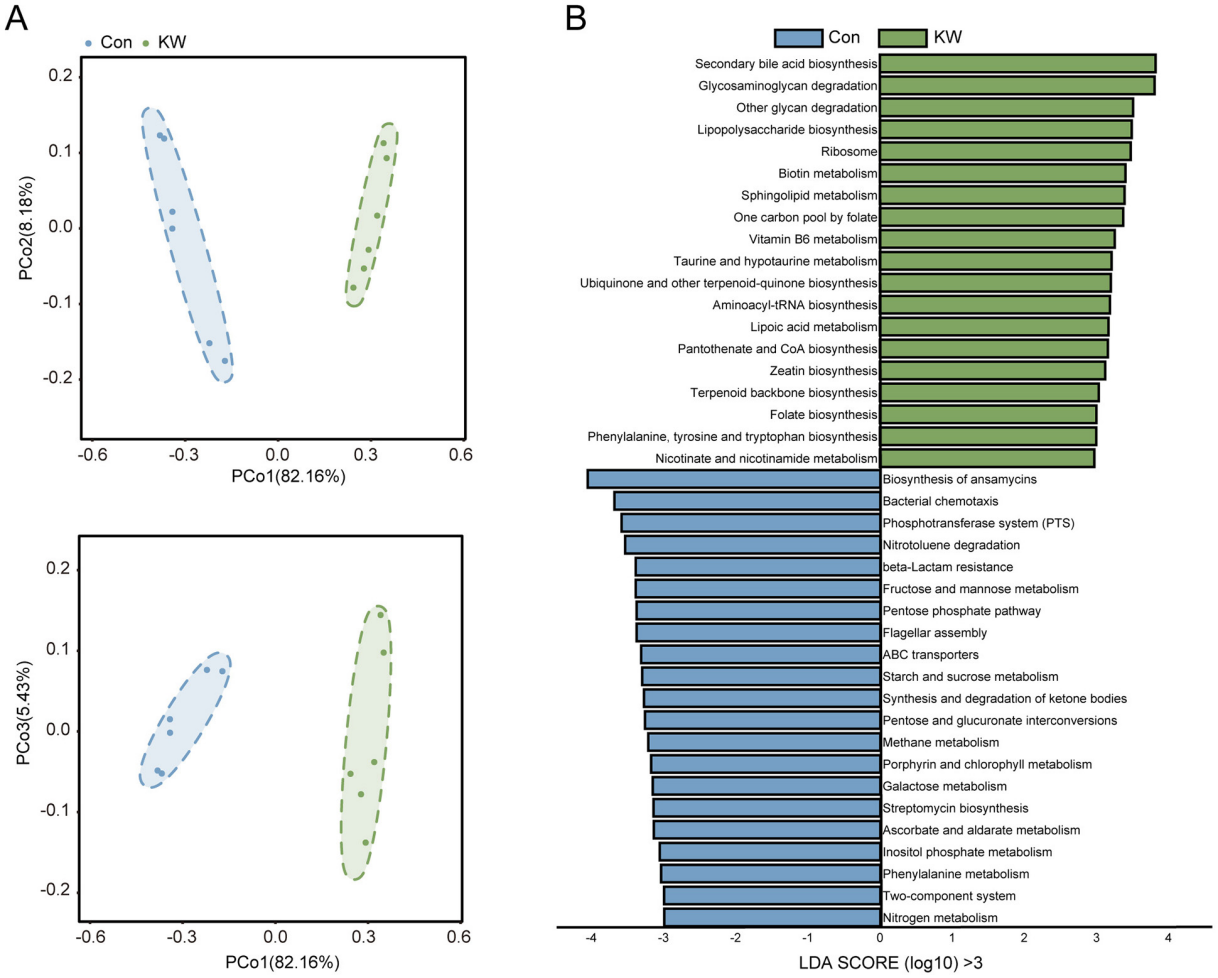
KW drinking significantly changed the metabolic pathways of the gut microbiota in mice **(A)** Principal Coordinates Analysis (PCoA) showed a distinct clustering of metabolic pathways between the two groups. *N* = 6. **(B)** The Linear Discriminant Analysis (LDA) score of the metabolic pathways with LDA Score (log_10_) greater than 3, indicating a higher abundance in the corresponding group. *N* = 6.

To analyze the functions of the gut microbiota, the functional PCoA showed a distinct clustering of microbial metabolic pathways between the two groups (Figure 5A), suggesting that the KW drinking induced a substantial shift in the function of the gut microbiota. Further pathway enrichment analysis showed that the following pathways were significantly enriched in KW group, including secondary bile acid biosynthesis, glycosaminoglycan degradation, sphingolipid metabolism, lipopolysaccharide biosynthesis, ribosome, biotin metabolism, other glycan degradation, one carbon pool by folate etc. (Figure 5B). In addition, the following pathways were significantly enriched in Con group, including biosynthesis of ansamycins, bacterial chemotaxis, phosphotransferase system, nitrotoluene degradation, fructose and mannose metabolism and beta-lactam resistance (Figure 5B). These findings suggest that KW consumption induces a functional reprogramming of the gut microbiota.

### KW drinking significantly decreased the basal level of inflammation

**Figure 6:**
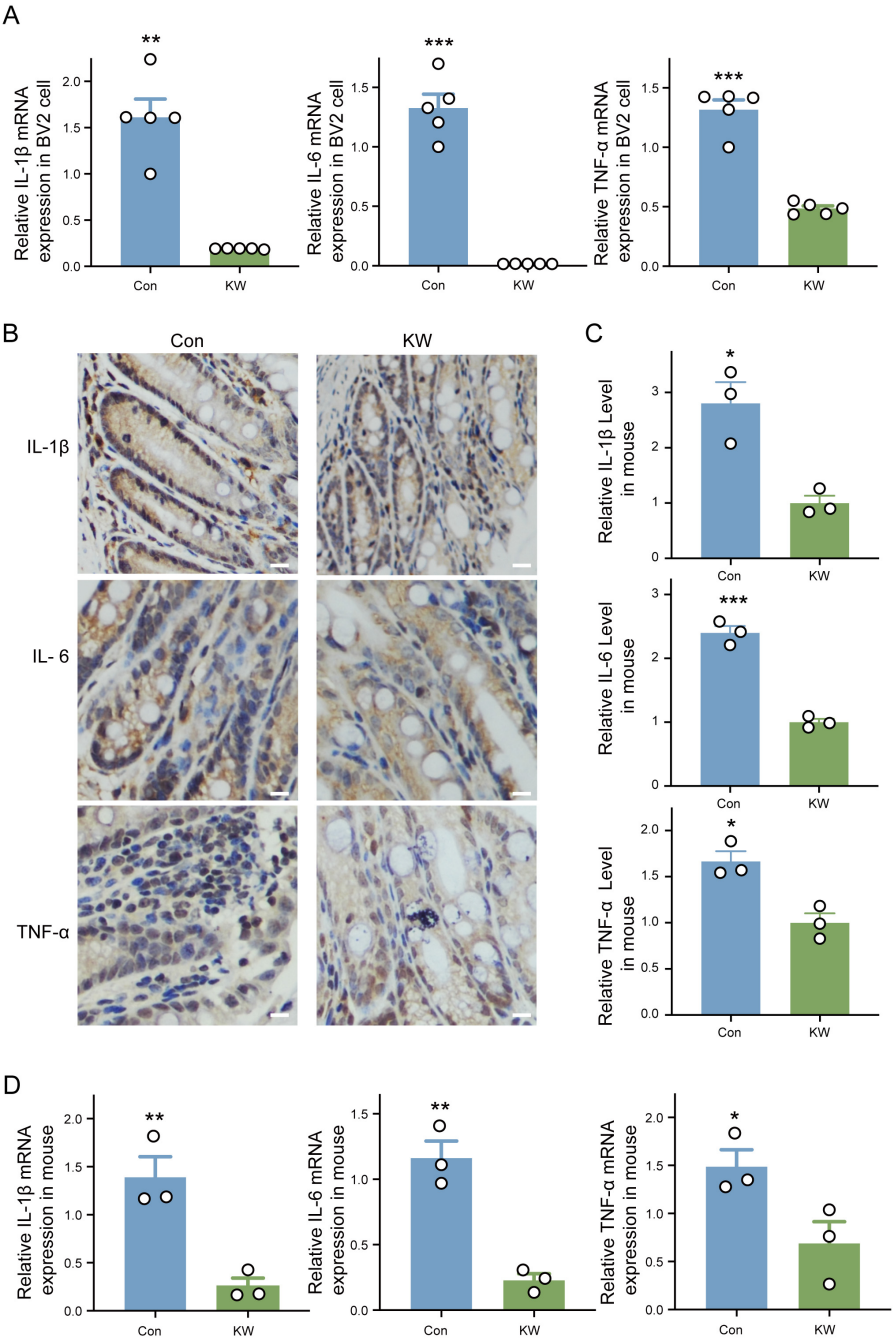
KW drinking significantly decreased the basal level of inflammation *in vitro* and *in vivo*. **(A)** The determination of *in vitro* basal mRNA levels of three major pro-inflammatory factors by real-time PCR, including IL-1β, IL-6, and TNF-α. N = 5. **(B, C)** Representative images and quantitative analysis of immunohistochemical analysis of the mouse colon. Scale bar = 10 μm, *N* = 3 **(D)** The determination of *in vivo* basal mRNA levels of three major pro-inflammatory factors by real-time PCR, including IL-1β, IL-6, and TNF-α. *N* = 3. *p < 0.05, **p < 0.01, ***p < 0.001.

The KW drinking was found to significantly decrease the *in vitro* basal mRNA levels of three major pro-inflammatory factors, including IL-1β, IL-6, and TNF-α (Figure 6A). The immunohistochemical analysis of mice colon indicated that KW drinking significantly decreased the *in vivo* basal levels of three major pro-inflammatory factors, including IL-1β, IL-6, and TNF-α (Figures 6B, C). Moreover, the real-time PCR also supported that KW drinking significantly decreased the *in vivo* basal levels of the three major pro-inflammatory factors (Figure 6D). The results suggested that KW drinking significantly decreased the basal level of inflammation.

## Discussion

Our study has obtained the following novel findings: First, KW was pure water containing ultra-small nanobubbles; second, KW drinking significantly increased the diversity and richness of the gut microbiota of the mice; third, KW drinking significantly changed the relative abundance of several phyla in the gut microbiota of mice; fourth, KW drinking increased significantly the abundance of several beneficial genera of the gut bacterium and decreased significantly the abundance of several harmful genera of the gut bacterium of mice; and fifth, KW drinking significantly changed the metabolic pathways of the gut microbiota in mice. Moreover, KW drinking was safe both *in vitro* and *in vivo*, and significantly decreased the basal level of three major pro-inflammatory factors including IL-1β, IL-6, and TNF-α.

### Physicochemical properties and antioxidant potential of KW

Our previous study was the first to demonstrate that KW enhances cellular antioxidant capacity (19). In this study, consistent with the manufacturer's report, we confirmed the presence of ultra-small nanobubbles in KW through the NTA system and DLS instrument. Previous studies have shown that ultra-small nanobubble water exhibits antioxidant activity (31). It might partially explain the underlying mechanism of the antioxidative effects of KW. It has also been reported that nanobubble water influenced the composition of gut microbiota in metagenomic insights (32). These studies support and are consistent with our findings: KW could enhance cellular antioxidant capacity and modulate gut microbiota. In addition, the heavy metal elements, pH, and TDS were determined to support that there were only ultra-small nanobubbles without any additives. Compared with other commercial pure water and mineral water, KW demonstrated an extremely high level of purity, which showed 0 ppm TDS and the closest pH to ddH_2_O.

### Enhanced microbial diversity and ecological stability

Our study verified the hypothesis that KW could modulate the gut microbiota, via a significant increase in the abundance of Verrocomicrobiota, and a decrease in the abundance of Firmicutes, and Proteobacteria. Ecological theory predicts that species-rich communities are less susceptible to invasion, suggesting that higher microbial richness and diversity enhance the environmental resilience of gut microbiota (33). Conversely, low microbial richness and diversity correlate with obesity, IBD, and *C. difficile*-associated disease (34–37). Our study has found that KW drinking led to significant increases in the richness and diversity of the gut microbiota, suggesting that KW drinking can also produce beneficial effects by increasing the richness and diversity of the gut microbiota.

### Remodeling of key microbial taxa

KW consumption was associated with the enrichment of several taxa with well-established beneficial effects, including *Akkermansia muciniphila (A.muciniphila), Faecalibacterium, Ligilactobacillus*, and *Muribaculum*. A number of studies have indicated that *A. muciniphila* is a beneficial gut bacterium that can produce multiple beneficial biological effects, including decreased inflammation, improved glucose metabolism, and decreased body fat (38). There is a higher abundance of *A. muciniphila* in the gut of healthy individuals, compared with that of individuals with metabolic disorders (38). *Faecalibacterium* is one of the critical types of bacteria in the human gut, which has multiple beneficial biological functions, such as anti-inflammatory function and production of n-Butyric acid. Both experimental and epidemiological data have indicated the great potential of *Faecalibacterium* for becoming a promising probiotic or live biotherapeutic product (39). Increasing evidence has also indicated that *Ligilactobacillus murinus*, a member of the *Ligilactobacillus* genus, plays beneficial roles in intestinal metabolism and immune activities of the host (40). There is also a close correlation between the abundance of *Ligilactobacillus murinus* and intestinal health, suggesting its significant potential as a probiotic (40). It has been indicated that *Muribaculum* plays beneficial roles in the metabolism of short-chain fatty acids (SCFA), polysaccharides, and cholesterol (41–44).

By contrast, KW significantly reduced harmful taxa such as *Clostridioides difficile* (*C. difficile*), *Escherichia-Shigella*, and *Citrobacter*, which are associated with gut barrier disruption and infection. *C. difficile* is the etiological agent for *C. difficile* infection (CDI), which is an antibiotic-associated diarrhea that can be fatal if untreated (45). *C. difficile* has become an “Urgent Threat” to the U.S. healthcare, since there is an annual CDI burden of approximately 220,000 cases and 13,000 deaths (45). *Shigella* belongs to the harmful gut microbiome, which is causative of bacillary dysentery in humans (46). Bacillary dysentery is characterized by invasion and inflammatory destruction of the human colonic epithelium (46). *Citrobacter* is a distinct group of human pathogens, members of which can cause neonatal meningitis and possibly gastroenteritis in children and adults (47). *Clostridium innocuum* is both part of the normal intestinal flora and the cause of an intrinsically vancomycin-resistant opportunistic infection in immunocompromised patients (48). *Clostridium innocuum* is also a vancomycin-resistant pathogen that may be causative of antibiotic-associated diarrhea (49). It has been reported that *Clostridium innocuum* is an opportunistic pathogen that is causative of progesterone resistance in women taking progesterone (50).

Besides the specific beneficial and harmful gut microbiota, we also found the changed abundance of some gut microbiota, including *Blautia, Marvinbryantia, Gastranaerophilales, and Bacteroides*. *Blautia* can play both beneficial and harmful biological roles: A number of studies have indicated that *Blautia* can play harmful roles in multiple conditions, while *Blautia* is also involved in the metabolism of SCFA (51–54). Our study has also shown that the KW drinking led to a dramatic decrease in the abundance of *Marvinbryantia* in the gut microbiota. It has been indicated that *Marvinbryantia* can have both harmful and beneficial effects under different conditions, e.g., the herbal medicine Berberidis Cortex produced significantly decreased levels of pro-inflammatory cytokines, when it significantly decreased the abundance of *Marvinbryantia* (55). In contrast, *Marvinbryantia* showed protective effects for vitiligo (56). Our study also found that the KW drinking led to an increased abundance of *Gastranaerophilales*. It has been indicated that *Gastranaerophilales* can play either beneficial roles or harmful roles under different conditions. There is evidence suggesting that *Gastranaerophilales* may produce beneficial effects (57, 58). However, in a study on the effect of hawthorn seed oil on the plasma level of cholesterol and the gut microbiota of hypercholesterolemia hamsters, it was found that SCFA production was negatively correlated with the abundance of *Gastranaerophilales* (59). Our study found that the KW drinking led to an increased abundance of *Bacteroides. Bacteroides* belong to a genus of gut bacterium that can produce both beneficial effects and harmful effects: (60) *Bacteroides* are critical players in the maintenance of eubiosis of the human gastrointestinal tract, which can produce SCFA, including butyrate, producing various beneficial effects on the host; *Bacteroidota* can also contribute to colonization-resistance of harmful organisms and maintenance of gut integrity. However, some *Bacteroides spp*. can become opportunistic pathogens in multiple conditions, such as intra-abdominal infection.

### Functional metabolic reprogramming

The gut microbiota affects the physiological and pathophysiological states of the host through multiple mechanisms and is widely involved in the regulatory processes of various diseases, which is crucial for maintaining the homeostasis of the body (61). Based on the Lefse analysis, significant differences in Kyoto Encyclopedia of Genes and Genomes (KEGG) metabolic pathways were observed between the Con group and the KW group. In the KW group, first, intestinal barrier integrity and nutrient metabolism were significantly enhanced via secondary bile acid biosynthesis, sphingolipid metabolism, and glycosaminoglycan degradation. Bile acids are key signaling molecules that mediate the dialogue between the host and intestinal microbiota (62). They influence various physiological functions of the host, such as glycolipid metabolism and immune response, by regulating host receptors like Farnesoid X Receptor (FXR), TGR5, and Vitamin D Receptor (VDR). A recent study has reported that metabolic dysfunction-associated steatohepatitis could be alleviated by a secondary bile acid biosynthetic pathway (63). Glycosaminoglycans (GAGs) are important components of the extracellular matrix (ECM) and the intestinal barrier, playing a role in immune regulation, cell communication, and tissue repair (64). Glycosaminoglycan degradation produces short-chain fatty acids, enhances intestinal barrier function, and reduces intestinal permeability (64). Sphingolipids are synthesized as signal lipids to maintain cell membrane structure and immune regulation. The sphingolipid metabolites, ceramide, ceramide-1-phosphate (C1P), and sphingosine-1-phosphate (S1P) have been regarded as important signaling molecules that regulate cell growth, immune cell trafficking, and vascular integrity (65). The enriched Sphingolipid metabolism suggested the KW could maintain the intestinal barrier and enhance anti-inflammatory capacity. Second, vitamin and coenzyme metabolism-related pathways were enriched in the KW group, including biotin metabolism, vitamin B6 metabolism, one-carbon pool by folate, folate biosynthesis, pantothenate and CoA biosynthesis, lipoic acid metabolism, and nicotinate and nicotinamide metabolism. These pathways alleviate inflammatory levels, reduce oxidative stress, enhance immunity, and improve energy metabolism. Finally, several metabolic pathways enriched in the KW group suggested KW drinking could promote cell repair capability, such as aminoacyl-tRNA biosynthesis, ubiquinone and other terpenoid-quinone biosynthesis, and terpenoid backbone biosynthesis. The metabolic pathways in the Con group are enriched in antibiotic biosynthesis (biosynthesis of ansamycins, streptomycin biosynthesis, biosynthesis of vancomycin group antibiotics), bacterial chemotaxis and flagellar assembly, and high carbohydrate metabolism activity (fructose, galactose, starch), along with stress-response and antibiotic resistance-related systems, such as two-component systems and beta-lactam resistance. These features may reflect a baseline microbial functional profile that, when compared with the KW group, shows relatively greater enrichment of chemotaxis, carbohydrate metabolism, and stress-associated pathways.

### Reduction of basal inflammation

A number of studies have indicated that gut microbiota plays a crucial role in both inflammation in the intestinal barrier and systemic inflammation: (66) First, under healthy conditions, a low level of basal inflammation is persistent in the intestinal barrier, while dysregulation of gut microbiota can intensify inflammation in the intestinal barrier; second, metabolites from gut microbiota can produce profound effects on intestinal barrier and systemic inflammation: such metabolites from gut microbiota as SCFA and bile acids can produce anti-inflammatory effects, while such metabolites from gut microbiota as lipopolysaccharides (LPS) can increase inflammatory responses; third, such factors as dietary habitats or aging can lead dysregulation of gut microbiota, leading to such pathological changes as metabolic endotoxemia and serum inflammation; and fourth, gut viral particles can be released into the lumen under certain conditions, which could penetrate through the gut epithelium leading to exacerbated immune responses. Consistent with these established mechanisms, the microbial shifts induced by KW drinking in our study align well with the observed reductions in basal inflammatory cytokines. Several genera that increased in the KW group, including *Akkermansia, Faecalibaculum, Ligilactobacillus*, and *Muribaculum*, are widely reported to enhance mucosal barrier function, promote SCFA production, and attenuate inflammatory signaling pathways (38–41). Conversely, genera that decreased following KW consumption, such as *Escherichia-Shigella, Clostridioides*, and *Citrobacter*, are known contributors to endotoxin release, epithelial disruption, and pro-inflammatory responses (45–47). In addition, the KW-enriched metabolic pathways, including secondary bile acid biosynthesis, sphingolipid metabolism, glycosaminoglycan degradation, and folate-related pathways, have been closely associated with anti-inflammatory and barrier-protective functions (62–65). Hence, KW can reduce the basal inflammatory levels by modulating the gut microbiota composition.

Since our study has indicated that KW drinking can produce significant beneficial effects on the gut microbiota of the mice, we tested our hypothesis that KW drinking may also lead to a decrease in the basal level of inflammation in the mice. Our study has found that KW drinking produced significant decreases in the three major pro-inflammatory factors, including IL-1β, IL-6, and TNF-α, thus providing evidence supporting our hypothesis. These three pro-inflammatory factors are widely recognized as biomarkers of low-grade chronic diseases, even in the absence of overt clinical symptoms (67, 68). Sustained elevation of these cytokines is closely associated with a range of deleterious outcomes, including inflammaging, metabolic disorders, neurodegeneration, and cancer development (68, 69). Despite its clinical significance, chronic inflammation remains difficult to treat due to its subclinical state, systemic distribution, and the lack of safe, long-term therapeutic options.

Our study demonstrates that KW consumption can reduce basal inflammatory levels both directly and indirectly by modulating the gut microbiota composition in mice and promoting a more anti-inflammatory microbial profile. Given its excellent safety, affordability, and potential for daily use, KW might be suggested as a feasible strategy for low-grade chronic inflammation and the prevention of its associated pathological sequelae.

### Safety and translational implications

Finally, we evaluated the safety of the KW intervention using a comprehensive set of cellular, physiological, and histological parameters. No significant differences were observed between the KW and Con groups in terms of cell viability (Figure 2C), extracellular and intracellular LDH release (Figure 2D), intracellular ROS generation (Figures 2E, F), or NO production (Figure 2G), indicating that KW treatment did not induce cytotoxicity or oxidative stress *in vitro*. *In vivo* assessments further confirmed the biocompatibility of KW. No significant differences were detected in organ morphology (Figure 2I), histological architecture (Figure 2J), body weight trajectory (Figure 2K), or DAI (Figure 2L) throughout the experimental period. Additionally, organ indices, including colon length (Figure 2M), spleen, heart, liver, lung, and kidney index (Figures 2N–R), were comparable between the two groups, suggesting no signs of systemic toxicity or organ burden.

Collectively, based on the previously confirmed significant antioxidant activity of KW water, our current study confirmed that it can modulate the gut microbiota and both directly and indirectly decrease the basal level of inflammation (19). Given the importance of low-grade chronic inflammation, in-depth studies are required to better understand the mechanistic basis and translational applications of KW as a potential intervention. In addition, the precise mechanisms by which KW modulates gut microbiota composition remain to be fully elucidated. It is of great interest to investigate the mechanisms underlying the effects of KW drinking on the gut microbiota of mice. In light of its demonstrated antioxidant capacity, anti-inflammatory effects, and ability to modulate gut microbiota, KW holds promise for broader biomedical applications that warrant further investigation.

## Conclusions

Our study has obtained novel evidence that KW is capable of both improving the gut microbiota and decreasing the basal level of inflammation in mice. The significantly increased abundance of several beneficial genera (*Akkermansia, Faecalibaculum, Ligilactobacillus*, and *Muribaculum*) and significantly decreased abundance of several harmful genera (*Clostridioides, Citrobacter, Escherichia-Shigella*, and *Clostridium Innocuum group*) of the gut bacterium strongly supported the improvement of KW. In addition, KW drinking showed the dramatic enrichment of beneficial microbial metabolic pathways closely associated with enhanced mucosal barrier integrity, host metabolic regulation, and antioxidative capacity. In summary, we identified a functional water that, characterized by its affordability, safety, and suitability for daily consumption, can improve metabolic function by modulating the gut microbiota and ultimately reduce the basal level of inflammation. These findings suggest its potential as a non-pharmacological intervention for low-grade chronic inflammation under non-pathological conditions.

## Data Availability

The original contributions presented in the study are publicly available. The 16S rRNA gene sequencing data have been deposited in the NCBI Sequence Read Archive (SRA) under the BioProject accession number PRJNA1389860. This data can be found here: https://www.ncbi.nlm.nih.gov/sra/PRJNA1389860.
