# Supplementary material for: A novel functional water significantly modulates the gut microbiota and decreases the basal level of inflammation in mice
Source: Front Nutr. 2026 Jan 12;12:1718745. doi: 10.3389/fnut.2025.1718745 (PMC12832262; doi:10.3389/fnut.2025.1718745)
Supplement: Supplementary file 1 [file Table_1.docx]

**Supplementary Information**

**Fig. S1 The industrial preparation process of KW (A)**

**
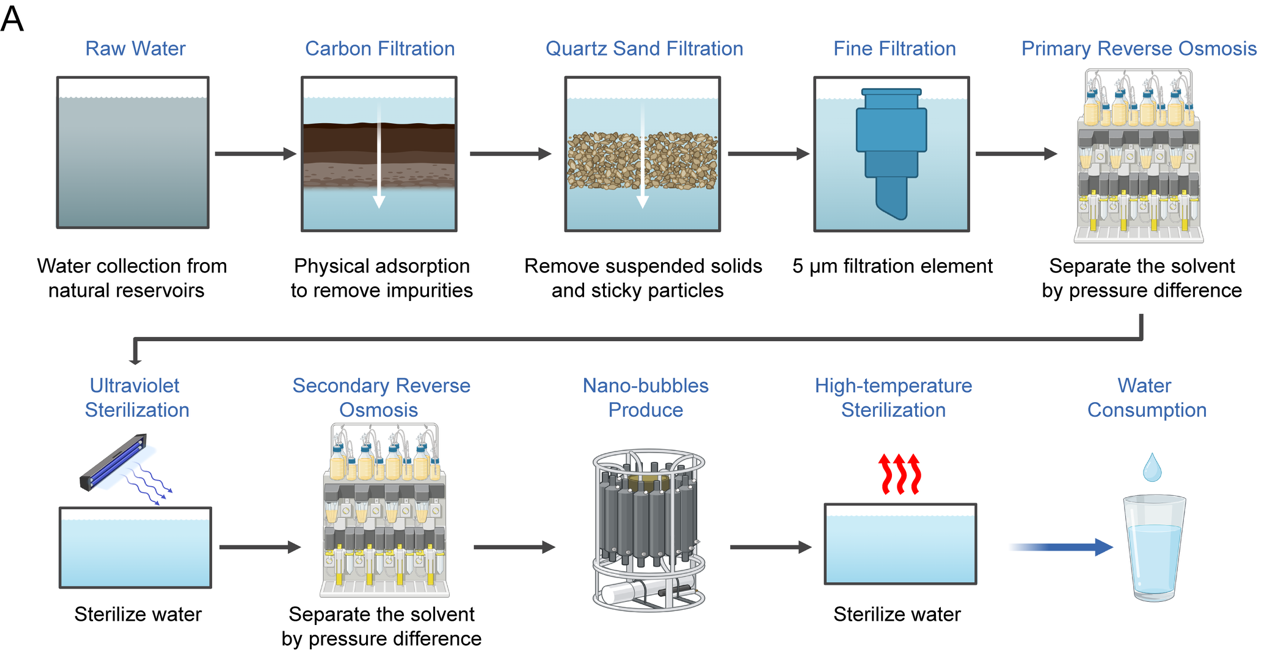
**

**Table S1. The raw data of heavy metal elements detection**

|  | Tap water | ddH_2_O | KW | Purified Water | Mineral Water 1 | Mineral Water 2 |
| --- | --- | --- | --- | --- | --- | --- |
| pH | 8.4 | 7 | 6.8 | 7.2 | 7.6 | 7.2 |
| Hardness mg/L | 25 | 0 | 0 | 0 | 0 | 0 |
| Mercury mg/L | 0 | 0 | 0 | 0 | 0 | 0 |
| Iron mg/L | 0 | 0 | 0 | 0 | 0 | 0 |
| Copper mg/L | 0 | 0 | 0 | 0 | 0 | 0 |
| Lead mg/L | 0 | 0 | 0 | 0 | 0 | 0 |
| Manganese mg/L | 0 | 0 | 0 | 0 | 0 | 0 |
| Total chlorine mg/L | 0 | 0 | 0 | 0 | 0 | 0 |
| Free chlorine mg/L | 0 | 0 | 0 | 0 | 0 | 0 |
| Nitrate mg/L | 10 | 0 | 0 | 0 | 0 | 0 |
| Nitrite mg/L | 1 | 0 | 0 | 0 | 0 | 0 |
| Zinc mg/L | 5 | 0 | 0 | 0 | 0 | 0 |
| Chromium/Cr (VI) | 0 | 0 | 0 | 0 | 0 | 0 |
| Sulfide mg/L | 0 | 0 | 0 | 0 | 0 | 0 |
| Sulfate mg/L | 0 | 0 | 0 | 0 | 0 | 0 |
| Fluoride mg/L | 0 | 0 | 0 | 0 | 0 | 0 |

**Table S2. The raw data of total dissolved solids**

|  | Tap water | ddH_2_O | KW | Purified Water | Mineral Water 1 | Mineral Water 2 |
| --- | --- | --- | --- | --- | --- | --- |
| pH | 148 | 0 | 0 | 0 | 25 | 38 |
| Hardness mg/L | 156 | 0 | 0 | 0 | 25 | 37 |
| Mercury mg/L | 157 | 0 | 0 | 0 | 23 | 38 |
| Iron mg/L | 149 | 0 | 0 | 0 | 26 | 35 |
| Copper mg/L | 151 | 0 | 0 | 0 | 24 | 39 |
| Lead mg/L | 156 | 0 | 0 | 0 | 27 | 37 |

**Table S3. ﻿Criteria for DAI scores**

| Score | Body weight loss (%) | Diarrhea | Bleeding  (Occult blood test) |
| --- | --- | --- | --- |
| 0 | ﻿≤ 1 | ﻿Normal | ﻿Negative |
| 1 | ﻿1-5 | ﻿- | ﻿- |
| 2 | ﻿6-10 | ﻿Softer stool | ﻿Positive |
| 3 | ﻿11-15 | ﻿- | ﻿- |
| 4 | ﻿≥ 15 | ﻿Watery stool | ﻿Visual blood |
